# Supplementary material for: The Long-Term DEHP Exposure Confers Multidrug Resistance of Triple-Negative Breast Cancer Cells through ABC Transporters and Intracellular ROS
Source: Antioxidants (Basel). 2021 Jun 11;10(6):949. doi: 10.3390/antiox10060949 (PMC8230873; doi:10.3390/antiox10060949)
Supplement: Supplementary file 1 [file antioxidants-10-00949-s001.zip › antioxidants-1239184-supplementary.pdf]

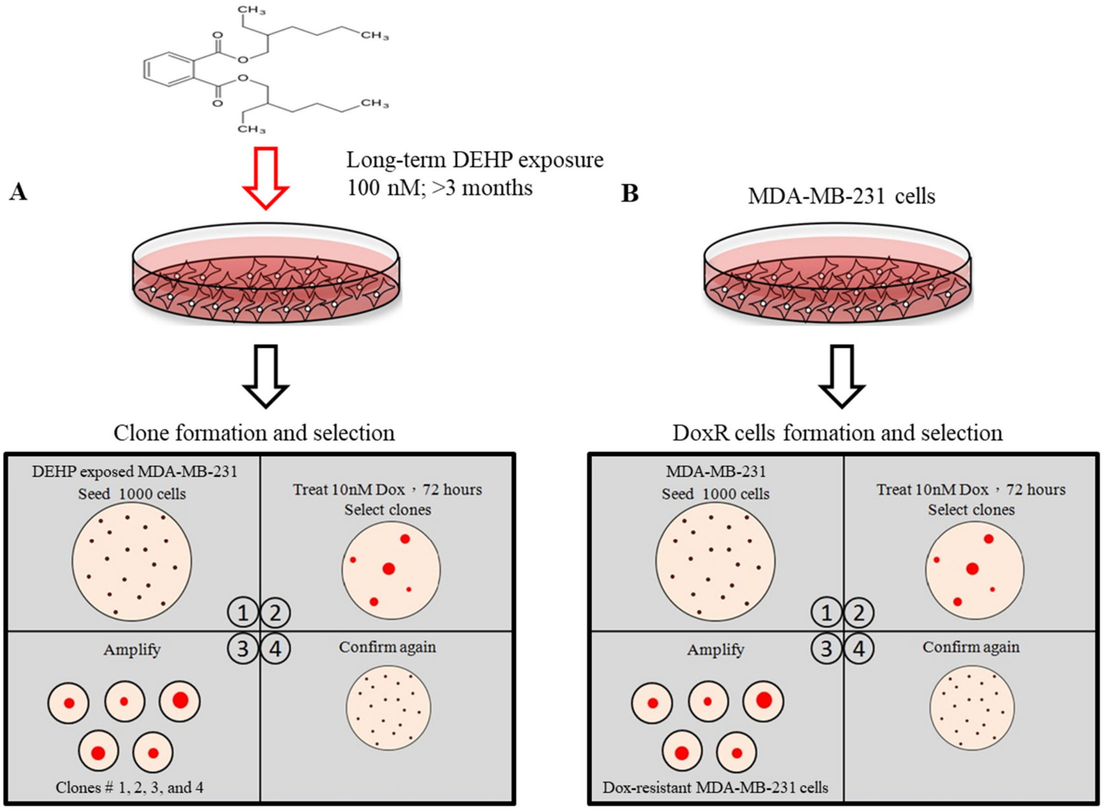

Figure S1. Schematics of DEHP exposure and establishment of stable drug resistant clones. **(A)** MDA-MB-231 cells were exposed to 100 nM DEHP for as long as 3 months. After exposure, DEHP was removed, and the cells were challenged with Dox (10 nM concentration for 72 hours). Dox-resistant colonies were selected, amplified, and maintained in culture medium for further investigations (referred to as clones # 1, 2, 3, and 4). **(B)** MDA-MB-231 cells were exposed to Dox (10 nM for 72 hours), cultured for 10 days to form colonies. Further, Dox resistant colonies were selected and maintained as DoxR cells.

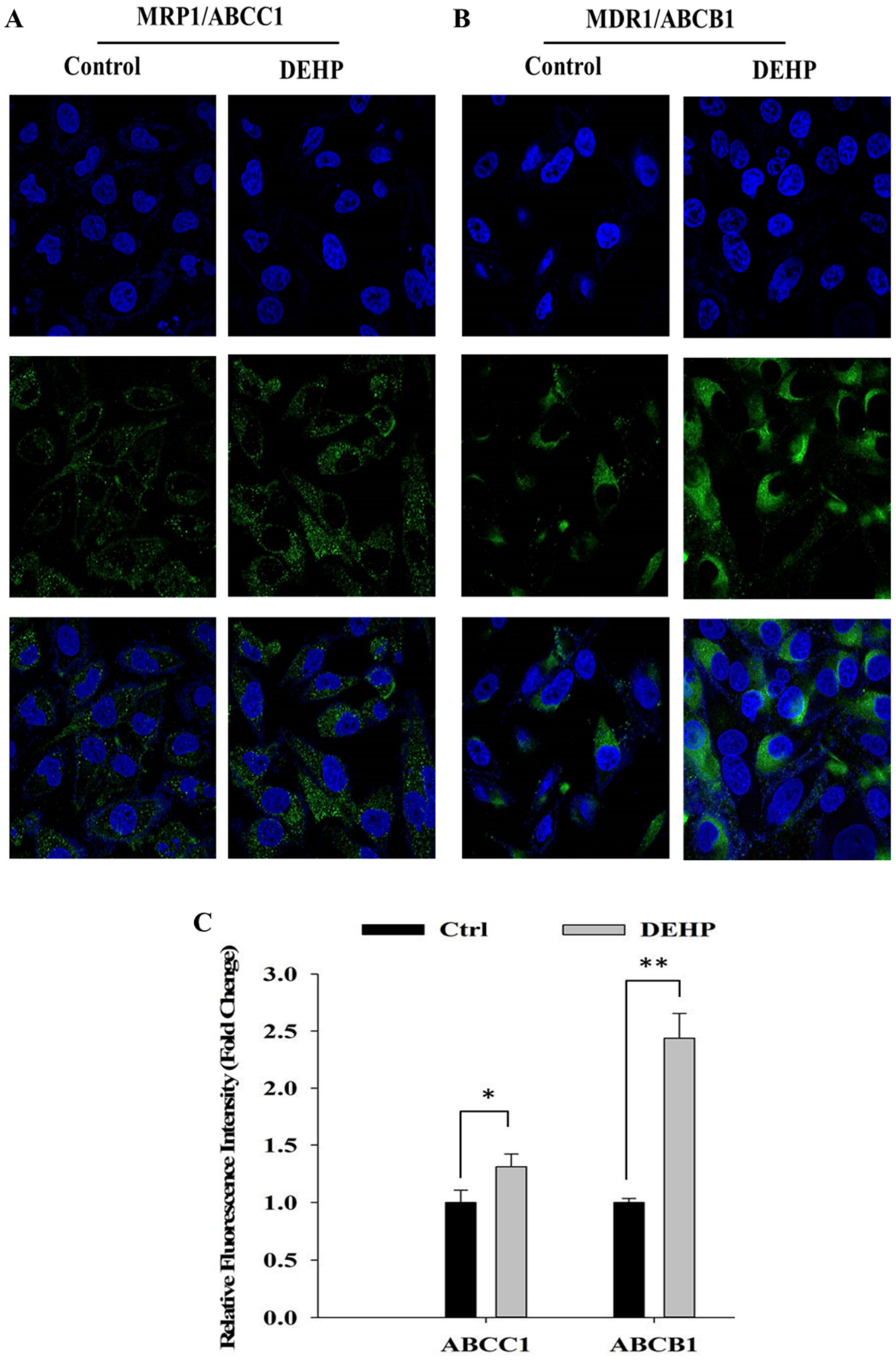

Figure S2. Representative and quantitative results of ABC transporters expression by immunofluorescence (IF). **(A) and (B)** Increased expression of MRP1/ABCC1 and MDR1/ABCB1 was observed in long term DEHP exposed clone, Green fluorescence - FITC conjugated antibody; blue fluorescence - DAPI (nuclear staining) as observed using an Olympus FV1000 confocal laser scanning microscope. **(C)** Quantitative analysis of fluorescence intensity. **p <0.001, * *p* <0.05.

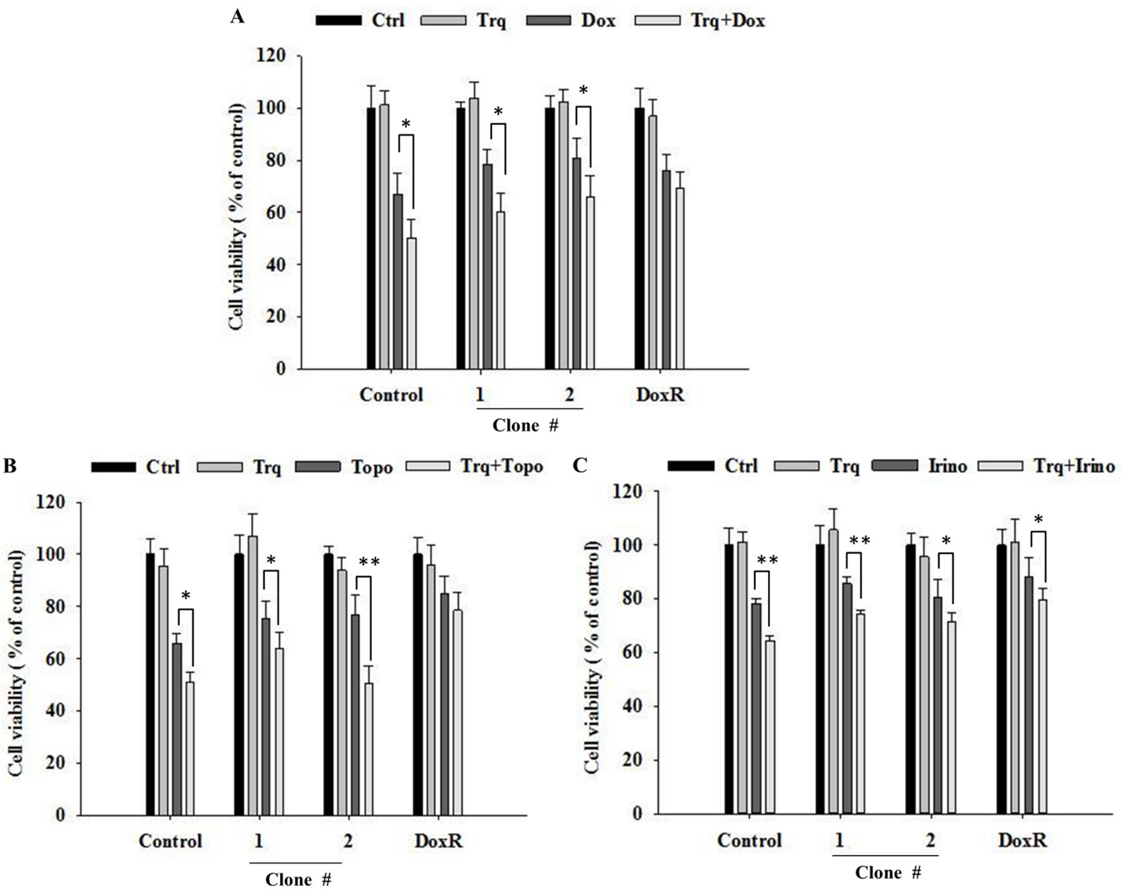

Figure S3. Quantitative analysis of cell viability by MTT assay. Control, Dox-resistant and DEHP-exposed MDA-MB-231 clones (# 1 & #2) show induced cell viability against Dox (1 μM), topotecan (1 μM), and irinotecan (1 μM) for 24 hours compared to parental MDA-MB-231 cells; 2-hour tariquidar (P-gp inhibitor) pretreatment enhanced the drug cytotoxicity of **(A)** Dox, (B) topotecan, and **c.** irinotecan reversing the acquired drug resistance in long-term DEHP-exposed clones and parental MDA-MB-231 cells.

A

B

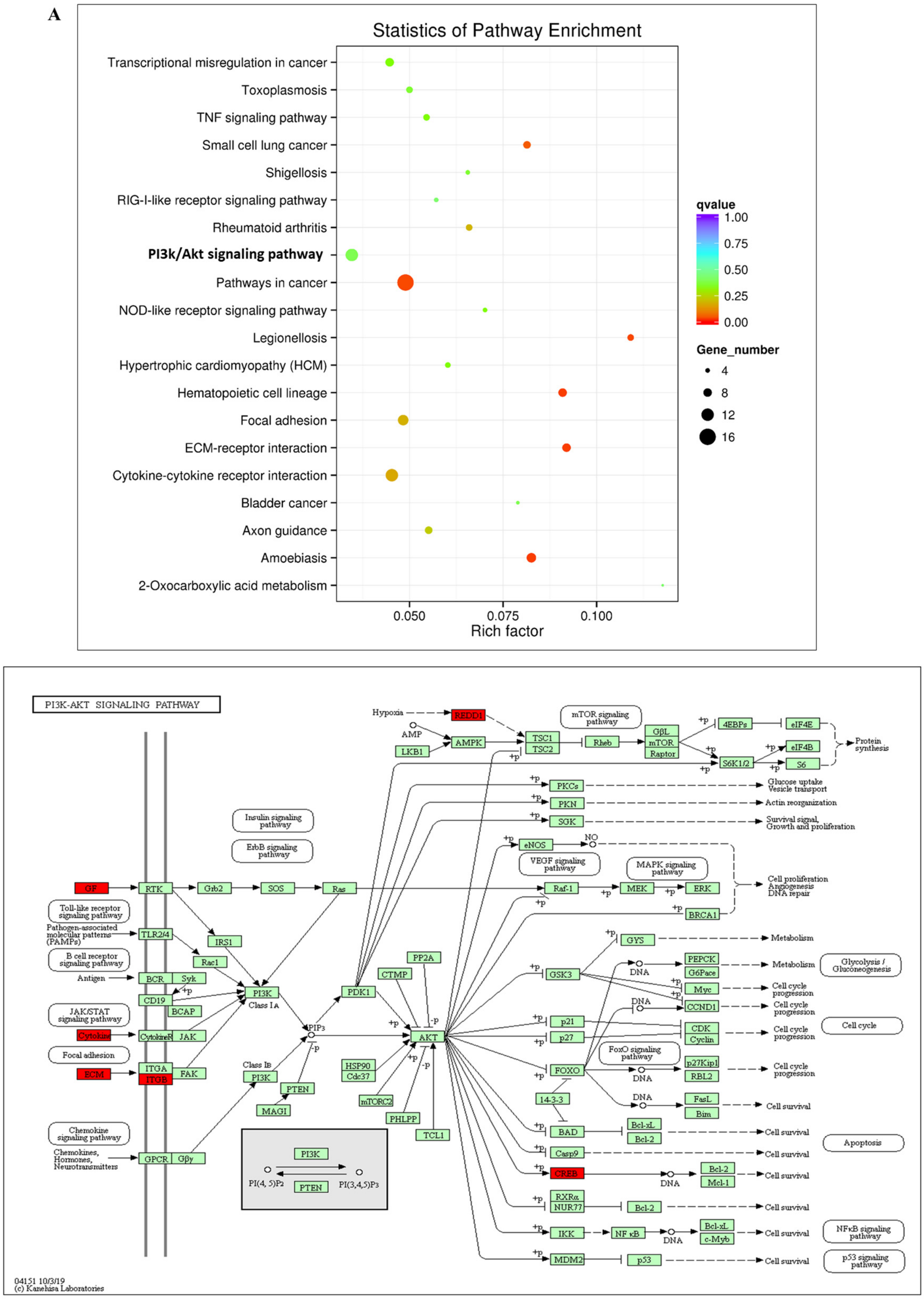

Figure S4. KEGG analysis of NGS data. (**A**). Significantly enriched KEGG pathways shown in a scatterplot. The scatterplot represents the top 20 significantly enriched pathways. The X-axis represents the detected pathways, and the Y-axis represents the rich factor. The size represents the number of different genes, and the color represents q values. **(B)** Differentially expressed genes in PI3K/Akt signaling by KEGG pathway analysis. Overall, the PI3K/Akt pathway was upregulated, ▪ red highlight shows specific gene upregulation in the pathway.
